# Accelerated biological aging as potential mediator in the relationship between central obesity and lung cancer risk

**DOI:** 10.3389/fragi.2025.1667490

**Published:** 2025-09-19

**Authors:** Hongya Liu, Zhihao Deng, Zhongwen Gong, Yangjiao Bai, Yongjia Li, Qing Zhou, Jian Ma, Jing Gao, Xuemei Lian

**Affiliations:** ^1^ Key Laboratory of Molecular Biology for Infectious Diseases (Ministry of Education), Center for Lipid Research, The Second Affiliated Hospital, Chongqing Medical University, Chongqing, China; ^2^ Department of Nutrition and Food Hygiene, College of Public Health, Chongqing Medical University, Chongqing, China

**Keywords:** obesity, lung cancer, biological aging acceleration, biological senescence, biological age

## Abstract

**Background:**

The increased prevalence of obesity and incidence of lung cancer have raised significant concerns worldwide. However, the relationship between obesity and lung cancer risk, and the potential mediating effect of biological aging remains poorly understood.

**Methods:**

Using UK Biobank database, this population-based cohort study employed multivariable Cox regression to estimate HRs (Hazard Ratios) for obesity indices (waist circumference [WC], waist-hip ratio [WHR], body shape index [ABSI], conicity index [C-Index]) and lung cancer risk. Biological aging was evaluated via PhenoAge and Klemera-Doubal method age (KDMAge), with acceleration calculated by regressing biological on chronological age. Longitudinal mediation analysis explored their mediating effects.

**Results:**

Among the 301,398 participants in the study, 2,466 incident cases of lung cancer were identified. All central - obesity - related indices were significantly associated with elevated risk of lung cancer, with (WC: HR = 1.10, 95% CI 1.02–1.19; WHR: 1.10, 1.03–1.18; ABSI: 1.73, 1.54–1.94; C-Index: 1.51, 1.35–1.69). Notably, PhenoAge/KDMAge acceleration mediated the associations between WHR, ABSI, C -Index and the lung cancer risk, with mediated proportions from 1.85% to 32.67%.

**Conclusion:**

This study highlights central obesity was significantly associated with incident risk of lung cancer, emphasizing biological aging’s mediating role.

## 1 Introduction

Lung cancer is the leading cause of cancer death worldwide, with an estimated 2.5 million new cancer cases and 1.8 million deaths in 2022 (Bray et al., 2024). Smoking is by far the main risk factor for lung cancer worldwide. However, other factors like environmental/occupational exposures, chronic obstructive pulmonary disease (COPD), family history, socioeconomic factors, and metabolic status also contribute to its incidence (Bade and Dela Cruz, 2020; Schwartz, 2024; Park et al., 2024; Leiter et al., 2023). Since approximately 30% of lung cancer cases would be overlooked when using current risk factors to identify high - risk groups for lung screening, finding additional etiological factors is crucial (Robbins et al., 2021).

Obesity raises risks for cancers like colorectal and liver, but its link to lung cancer risk is controversial. Meta - analyses showed a counter - intuitive link between high body mass index (BMI) and lower incidence, which be due to BMI’s limitations, smoking confounding, and reverse causation (Rubino et al., 2025; Vasiliki et al., 2024). In contrast, studies using waist circumference (WC), waist-hip-ratio (WHR) and a body shape index (ABSI) found an increased lung cancer risk with obesity, suggesting central obesity as a key risk factor (Vedire et al., 2023). Li et al. also reported MetS was linked to higher lung cancer risk using the UK Biobank cohort (Li et al., 2024). These point to obesity, especially central obesity and metabolic dysregulation, in lung cancer etiology, which needs to be further elucidated. Notably, the pathological hallmarks of obesity driving these associations (e.g., chronic inflammation, insulin resistance, oxidative stress) have increasingly been recognized as key drivers of accelerated biological aging (Terron-Puig et al., 2022; Sanada et al., 2018). While the link between obesity and lung cancer is gaining clarity, aging itself is another pivotal factor in cancer research that cannot be overlooked.

Aging, an extraordinarily intricate biological process entailing multi-system physiological dysregulation, markedly augment the susceptibility to a wide array of diseases including cancer (Park et al., 2024; Ferrucci et al., 2020). Although chronological age (CA) is a fundamental marker of aging, individuals with the same chronological age may experience varying rates of biological age (BA), the latter combines information from biological markers, which might better reflect an individual’s physiology and risks of age-related diseases and death (Jia et al., 2017). Klemera-Doubal method age (KDMAge) and phenotypic age (PhenoAge) are two commonly used BA metrics (Levine et al., 2018; Klemera and Doubal, 2006). They rely on a set of easily obtainable clinical measurements and blood test indicators to provide a comprehensive assessment of a person’s biological age. With the escalating concern on the health impacts of obesity, accumulating studies have indicated that obesity may be a critical factor for accelerating biological aging, but the complex relationship among central obesity and aging, and possibly lung cancer risk has not been identified.

In the present study, we prospectively explored the associations among central obesity, accelerated biological aging and the risk of lung cancer. We found that obesity not only increased the risk of lung cancer but also accelerated the process of biological aging. Moreover, the acceleration of biological age plays an important role in central obesity-associated lung cancer incident.

## 2 Materials and methods

### 2.1 Study design and population

The study population was derived from the UK Biobank, a population-based nationwide prospective cohort study as previously described in detail (Sudlow et al., 2015). Data were gathered via questionnaires, interviews, and measurements, and health outcomes were tracked via links to external datasets. The exclusion criteria included prevalent cancer (except nonmelanoma skin cancer) at baseline; incomplete BMI, WC, and hip circumference (HC) data; extreme WC and HC values; missing covariate data; and missing biological age-related indicator data. Finally, 301,398 participants were included (Supplementary Figure S1). The UK Biobank received approval from relevant boards and ethics committees, and all participants provided informed consent.

### 2.2 Central obesity measurement indicator assessment

The general physical examination included height, weight, WC and HC. From these primary measurements, three validated central obesity assessment indices were derived and computed in strict accordance with the formulas detailed below: WHR, ABSI, and conicity index (C-Index). Specifically, the C-Index is a specialized quantitative metric that evaluates the degree of central adiposity (i.e., abdominal fat accumulation) by quantifying the conical morphological characteristics of the human torso. Methodological foundations for these computations are referenced previous studies (Krakauer and Krakauer, 2012; Andrade et al., 2016).

$$WHR=\frac{WC\left(cm\right)}{HC\left(cm\right)}$$

$$ABSI=\frac{WC\left(cm\right)}{\left[{BMI\left(kg/m^{2}\right)}^{2/3}\times {Height\left(m\right)}^{1/2}\right]}$$

$$C-Index=0.109^{-1}\times WC\left(cm\right)\times {\left[\frac{Weight\left(cm\right)}{Height\left(cm\right)}\right]}^{-1/2}$$

Following the guidelines proposed by the World Health Organization in 2008 (WHO, 2008), the stratification are as follows: WC: normal (≤80 cm in women or ≤94 cm in man), increased (80–88 cm in woman or 94–102 cm in man), and substantially increased (>88 cm in woman and male>102 cm in woman). WHR: normal WHR (<0.85 for women or <0.9 for man) and substantially increased WHR (≥0.85 for women or ≥0.90 for man).

### 2.3 Biological aging assessment

KDMAge and PhenoAge, two clinical - parameter biological age algorithms, are validated and widely used, potentially representing different aspects of biological aging (Levine et al., 2018; Klemera and Doubal, 2006; Kuo et al., 2021). The process of calculation was described minutely elsewhere (Klemera and Doubal, 2006; Basu et al., 2018). KDMAge is calculated via regression, considering 9 clinical indicators: systolic blood pressure (SBP), total cholesterol, albumin, glycated hemoglobin (HbA1c), creatinine, C-reactive protein (CRP), alkaline phosphatase, blood urea nitrogen concentrations, and forced expiratory volume in 1 s (FEV1). PhenoAge was calculated based on ten variables: chronological age, albumin, creatinine, glucose, CRP, lymphocyte percent, mean cell volume, red blood cell distribution width, alkaline phosphatase, and white blood cell count (Levine et al., 2018). The Supplementary Material section contains the specific calculation formulas for PhenoAge and KDMAge. Biological age acceleration was calculated as the residual from a linear regression model in which biological age was regressed on chronological age. This residual represents the extent to which an individual’s biological age deviates from the expected biological age for their chronological age, with positive values indicating accelerated biological aging and vice versa (Basu et al., 2018).

### 2.4 Lung cancer assessment

Cancer diagnosis data in the UK Biobank were from national cancer and death registries. The study outcome was incident lung cancer (ICD–10 C33–C34). The endpoint was the first lung cancer diagnosis or the primary underlying cause of lung - cancer death, whichever was earlier. Participants were followed from enrollment until cancer diagnosis (excluding non - melanoma skin cancer), death, withdrawal from the cohort or the last follow - up date (30 September 2021 for England and Wales; 31 October 2021 for Scotland), whichever occurred first.

### 2.5 Covariates

The covariates included in this study were selected based on their relevance to lung cancer and refer to previous similar studies (Li et al., 2024). They include a range of demographic and health-related variables, age (continuous), gender (male/female), race/ethnicity (White, Mixed, Asian, Black, Others), education level (College/university, secondary, professional, none of above) and Townsend deprivation index (median - based), smoking status (never, former, current), alcohol drinker status (never, previous, current), history of hypertension/hyperlipidemia/diabetes (yes/no each), COPD was counted if self - reported by physician or spirometric criteria (FEV1/FVC < 0.7 and FEV1 < 80% predicted) met. Family history of lung cancer/diabetes/hypertension/hyperlipidemia (yes/no each). To rule out the robustness of biological age-related blood biochemical indicators, fasting time was added as a covariate.

### 2.6 Statistical analyses

Baseline characteristics were described using mean ± SD for continuous variables and frequency (proportion) for categorical variables. The t-test, Wilcox rank-sum test, and chi - square test were used where appropriate to compare baseline characteristics between groups with lung cancer and without lung cancer.

The proportional hazard assumption was verified via Schoenfeld residuals. Cox proportional hazards regression models estimated HRs and 95% CI for central obesity and the risk of lung cancer. Multivariable logistic regression analyzed the association between obesity and KDMAge, PhenoAge, reported as odds ratios (OR) and 95% CIs. Restricted cubic spline regression models were constructed to visualize the exposure–response curves between central obesity, KDMAge/PhenoAge, and lung cancer risk.

Stratified analyses were conducted by Gender (female, male) and smoking status (never, previous, currently). The effects of modifications between central obesity and Stratified factors on the risk of outcomes were examined via multivariable-adjusted Cox models (Model 2). Two sensitivity analyses ensured result robustness. All analyses were performed in R 4.2.2. A two - sided *P* < 0.05 was considered significant.

## 3 Results

### 3.1 Baseline characteristics of the study participants in the UK biobank

The baseline characteristics of the study participants was presented in Table 1. Among the 301,398 participants from the UK Biobank, 2,466 (0.82%) incident cases of lung cancer were identified over a median follow - up period of 11.72 years. At baseline, the mean age of the participants was 56.17 ± 8.10 years, and approximately 53% of them were female and 47% male. The mean values of KDMAge and PhenoAge for all participants were 52.81 ± 12.91 years and 56.89 ± 9.60 years, respectively.

**TABLE 1:** Baseline characteristics of the participants.

Characteristic	Overall (N = 301,398)	Nonlung cancer (N = 298,932)	Lung cancer (N = 2,466)	*P* value^a^
Survival Time, Mean (SD)	11.72 (2.66)	11.76 (2.61)	6.29 (3.35)	<0.001
Age, Mean (SD)	56.17 (8.10)	56.13 (8.10)	61.73 (5.77)	<0.001
Gender, n (%)				<0.001
Female	159,405 (53%)	158,249 (53%)	1,156 (47%)	
Male	141,993 (47%)	140,683 (47%)	1,310 (53%)	
Race, n (%)				<0.001
White	285,247 (95%)	282,855 (95%)	2,392 (97%)	
Mixed	1,794 (0.6%)	1,783 (0.6%)	11 (0.4%)	
Asian or Asian British	5,762 (1.9%)	5,744 (1.9%)	18 (0.7%)	
Black or Black British	4,075 (1.4%)	4,056 (1.4%)	19 (0.8%)	
Chinese	936 (0.3%)	928 (0.3%)	8 (0.3%)	
Other	3,584 (1.2%)	3,566 (1.2%)	18 (0.7%)	
Education, n (%)				<0.001
College or university degree	100,036 (33%)	99,619 (33%)	417 (17%)	
Secondary education	137,304 (46%)	136,296 (46%)	1,008 (41%)	
Some professional qualification	15,416 (5.1%)	15,288 (5.1%)	128 (5.2%)	
None of above	48,642 (16%)	47,729 (16%)	913 (37%)	
Townsend Deprivation Index, n (%)				<0.001
<−2.33	145,412 (48%)	144,497 (48%)	915 (37%)	
≥−2.33	155,986 (52%)	154,435 (52%)	1,551 (63%)	
Smoking, n (%)				<0.001
Never	167,364 (56%)	166,988 (56%)	376 (15%)	
Previous	103,404 (34%)	102,265 (34%)	1,139 (46%)	
Current	30,630 (10%)	29,679 (9.9%)	951 (39%)	
Alcohol drinker status, n (%)				<0.001
Never	12,511 (4.2%)	12,429 (4.2%)	82 (3.3%)	
Previous	9,968 (3.3%)	9,813 (3.3%)	155 (6.3%)	
Current	278,919 (93%)	276,690 (93%)	2,229 (90%)	
History of diabetes, n (%)				0.6
No	275,162 (91.3%)	272,903 (91.3%)	2,259 (91.6%)	
Yes	26,236 (8.7%)	26,029 (8.7%)	207 (8.4%)	
History of hypertension, n (%)				<0.001
No	122,337 (41%)	121,491 (41%)	846 (34%)	
Yes	179,061 (59%)	177,441 (59%)	1,620 (66%)	
History of hyperlipidemia, n (%)				<0.001
No	262,780 (87%)	260,806 (87%)	1,974 (80%)	
Yes	38,618 (13%)	38,126 (13%)	492 (20%)	
History of COPD, n (%)				<0.001
No	286,272 (95%)	284,363 (95.1%)	1,909 (77%)	
Yes	15,126 (5.0%)	14,569 (4.9%)	557 (23%)	
Family history of lung cancer, n (%)				<0.001
No	264,088 (88%)	262,163 (88%)	1,925 (78%)	
Yes	37,310 (12%)	36,769 (12%)	541 (22%)	
Family history of Hypertension, n (%)				<0.001
No	152,738 (51%)	151,249 (51%)	1,489 (60%)	
Yes	148,660 (49%)	147,683 (49%)	977 (40%)	
Family history of diabetes, n (%)				<0.001
No	234,262 (78%)	232,277 (78%)	1,985 (80%)	
Yes	67,136 (22%)	66,655 (22%)	481 (20%)	
Fasting Time, Mean (SD)	3.77 (2.39)	3.77 (2.39)	4.06 (2.79)	<0.001
WC, n (%)				<0.001
Normal	132,090 (44%)	131,206 (44%)	884 (36%)	
Increased	80,010 (27%)	79,327 (27%)	683 (28%)	
Substantially Increased	89,298 (30%)	88,399 (30%)	899 (36%)	
WHR, n (%)				<0.001
Normal	153,230 (51%)	152,336 (51%)	894 (36%)	
Substantially Increased	148,168 (49%)	146,596 (49%)	1,572 (64%)	
ABSI, n (%)				<0.001
Q1	75,352 (25%)	75,022 (25%)	330 (13%)	
Q2	75,348 (25%)	74,821 (25%)	527 (21%)	
Q3	75,349 (25%)	74,747 (25%)	602 (24%)	
Q4	75,349 (25%)	74,342 (25%)	1,007 (41%)	
C-Index, n (%)				<0.001
Q1	75,350 (25%)	74,998 (25%)	352 (14%)	
Q2	75,350 (25%)	74,808 (25%)	542 (22%)	
Q3	75,349 (25%)	74,755 (25%)	594 (24%)	
Q4	75,349 (25%)	74,371 (25%)	978 (40%)	
PhenoAge, Mean (SD)	56.89 (9.60)	56.83 (9.58)	64.35 (8.33)	<0.001
PhenoAge acceleration, Mean (SD)	0.71 (5.07)	0.70 (5.06)	2.62 (5.81)	<0.001
KDMAge, Mean (SD)	52.81 (12.91)	52.74 (12.89)	61.66 (11.91)	<0.001
KDMAge acceleration, Mean (SD)	−3.36 (10.23)	−3.39 (10.22)	−0.07 (10.57)	<0.001
PhenoAge acceleration, n (%)				
Non-PhenoAge acceleration	146,733 (49%)	145,869 (49%)	864 (35%)	<0.001
PhenoAge acceleration	154,665 (51%)	153,063 (51%)	1,602 (65%)	
KDMAge acceleration, n (%)				
Non-KDMAge acceleration	194,910 (65%)	193,650 (65%)	1,260 (51%)	<0.001
KDMAge acceleration	106,488 (35%)	105,282 (35%)	1,206 (49%)	

^a^ Wilcoxon rank sum test; Pearson’s chi-square test.

Abbreviations: WC (cm), waist circumference; WHR, waist–hip ratio; ABSI, body shape index; C-Index, conicity index; KDMAge, Klemera-Doubal method age; PhenoAge, phenotypic age.

### 3.2 Association of central obesity with the risk of lung cancer

**FIGURE 1:**
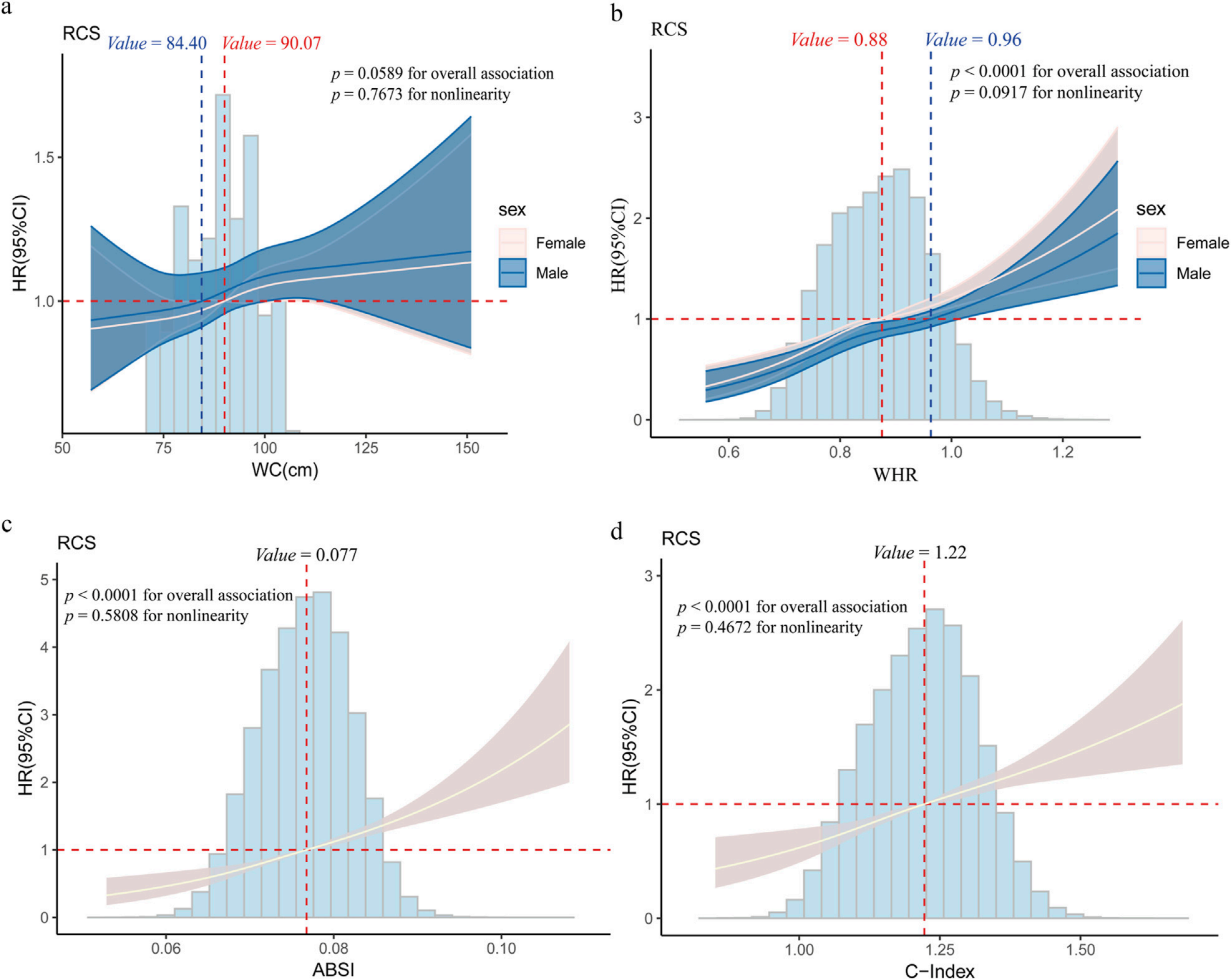
Restricted cubic spline (RCS) regression for the association between central obesity and lung cancer **(a)** Association between Waist Circumference (WC) and lung cancer risk; **(b)** Association between Waist-Hip Ratio (WHR) and lung cancer risk; **(c)** Association between A Body Shape Index (ABSI) and lung cancer risk; **(d)** Association between Conicity Index (C-index) and lung cancer risk. Age, gender, race, education level, the Townsend deprivation index, smoking status, alcohol drinker status, history of hypertension/hyperlipidemia/diabetes/COPD, and family history of lung cancer/diabetes/hypertension/hyperlipidemia were adjusted for associations. The cut-off values of WC and WHR were classified by gender (females are depicted by red dashed lines, males are depicted by blue dashed lines), and the cut-off values for the ABSI and C-Index were determined on the basis of the overall dataset. Abbreviations: WC (cm), waist circumference; WHR, waist–hip ratio; ABSI, body shape index; C-Index, conicity index.

After multivariable adjustment, significant positive linear associations were detected between WC, WHR, ABSI, and C-Index and incident lung cancer (*P* > 0.05, Figure 1). When WC ≥ 90.07 cm in females, ≥ 84.40 cm in males; WHR ≥ 0.88 in females, ≥ 0.96 in males; ABSI ≥ 0.077; and C Index ≥ 1.22, a positive linear correlation emerged. In the fully adjusted Model 2, the positive correlation between central obesity-related indices and the risk of lung cancer persisted (Table 2). The participants in the highest quartile of ABSI (HR = 1.73; 95% CI: 1.54–1.94) and the C-Index quartile (HR 1.51; 95% CI: 1.35–1.69) had higher lung cancer risk than did those in the lowest quartile. Furthermore, WC and WHR were linked to elevated risk, with HRs of 1.10 (95% CI: 1.02–1.19) for WC and 1.10 (95% CI: 1.03–1.18) for WHR.

**TABLE 2:** Association between central obesity and lung cancer.

Central obesity indices	Crude model		Model 1		Model 2	
	HR (95%CI)	*P* value	HR (95%CI)	*P* value	HR (95%CI)	*P* value
WC						
Normal	1 (ref)	—	1 (ref)	—	1 (ref)	—
Increased	1.24 (1.15–1.35)	<0.001	1.01 (0.93–1.09)	0.827	1.02 (0.94–1.11)	0.610
Substantially Increased	1.49 (1.39–1.61)	<0.001	1.09 (1.01–1.18)	0.023	1.10 (1.02–1.19)	0.015
*P* for trend		<0.001		0.033		0.321
WHR						
Normal	1 (ref)	—	1 (ref)	—	1 (ref)	—
Substantially Increased	1.40 (1.31–1.48)	<0.001	1.11 (1.04–1.18)	0.001	1.10 (1.03–1.18)	0.003
*P* for trend		<0.001		0.001		0.003
ABSI						
Q 1	1 (ref)	—	1 (ref)	—	1 (ref)	—
Q 2	1.59 (1.42–1.78)	<0.001	1.30 (1.16–1.45)	<0.001	1.2 (18.14–1.43)	<0.001
Q 3	1.96 (1.76–2.18)	<0.001	1.40 (1.24–1.56)	<0.001	1.37 (1.22–1.54)	<0.001
Q 4	3.38 (3.06–3.73)	<0.001	1.83 (1.63–2.05)	<0.001	1.73 (1.54–1.94)	<0.001
*P* for trend		<0.001		<0.001		<0.001
C-Index						
Q 1	1 (ref)	—	1 (ref)	—	1 (ref)	—
Q 2	1.53 (1.37–1.71)	<0.001	1.21 (1.08–1.32)	<0.001	1.19 (1.07–1.33)	0.002
Q 3	1.78 (1.60–1.98)	<0.001	1.22 (1.09–1.36)	<0.001	1.21 (1.08–1.35)	0.001
Q 4	3.03 (2.75–3.34)	<0.001	1.56 (1.39–1.74)	<0.001	1.51 (1.35–1.69)	<0.001
*P* for trend		<0.001		<0.001		<0.001

Crude model: Unadjusted.

Model 1: Adjusted for age, gender, race, Townsend deprivation index, education level, smoking status, and alcohol drinker status.

Model 2: Model 1 was further adjusted for history of hypertension, diabetes, hyperlipidaemia, COPD, family history of lung cancer, family history of diabetes, family history of hypertension, and family history of hyperlipidaemia.

Abbreviations: WC (cm), waist circumference; WHR, waist–hip ratio; ABSI, body shape index; C-Index, conicity index.

### 3.3 Analysis of the association between central obesity and biological age

The study revealed that central obesity-related indices were nonlinearly positively correlated with increased KDMAge acceleration and PhenoAge acceleration (*P* for nonlinearity <0.05, Supplementary Figure S2). Compared with those with a normal WC, those with a substantially increased WC demonstrated statistically significant increases in KDMAge acceleration (HR = 2.18; 95% CI: 2.14–2.22) and PhenoAge acceleration (HR = 2.28; 95% CI: 2.24–2.33; Table 3). Similarly, those with a markedly increased WHR, compared with those with a normal WHR, had a significant upwards trend in KDMAge acceleration (HR = 1.76; 95% CI: 1.73–1.80) and PhenoAge acceleration (HR = 1.48; 95% CI: 1.46–1.51). Compared with those in the lowest quartile, participants in the highest C-Index and ABSI quartiles had 1.50-year and 0.58-year increases in KDMAge acceleration, respectively. For PhenoAge acceleration, the highest C-Index and ABSI quartiles had 1.01-year and 0.30-year increases, respectively, compared with the lowest quartiles.

**TABLE 3:** Association between central obesity and biological aging.

Central obesity indices	Crude model		Model 1		Model 2	
	β (95%CI)	*P* value	β (95%CI)	*P* value	β (95%CI)	*P* value
WC and PhenoAge acceleration						
Normal	1 (ref)	—	1 (ref)	—	1 (ref)	—
Increased	1.36 (1.33–1.38)	<0.001	1.35 (1.32–1.37)	<0.001	1.34 (1.31–1.36)	<0.001
Substantially Increased	2.20 (2.16–2.24)	<0.001	2.33 (2.29–2.37)	<0.001	2.28 (2.24–2.33)	<0.001
*P* for trend		<0.001		<0.001		<0.001
WC and KDMAge acceleration						
Normal	1 (ref)	—	1 (ref)	—	1 (ref)	—
Increased	1.48 (1.46–1.51)	<0.001	1.48 (1.45–1.51)	<0.001	1.41 (1.38–1.44)	<0.001
Substantially Increased	2.49 (2.45–2.54)	<0.001	2.38 (2.33–2.42)	<0.001	2.18 (2.14–2.23)	<0.001
*P* for trend		<0.001		<0.001		<0.001
WHR and PhenoAge acceleration						
Normal	1 (ref)	—	1 (ref)	—	1 (ref)	—
Substantially Increased	1.92 (1.89–1.95)	<0.001	1.52 (1.50–1.54)	<0.001	1.48 (1.46–1.51)	<0.001
*P* for trend		<0.001		<0.001		<0.001
WHR and KDMAge acceleration						
Normal	1 (ref)	—	1 (ref)	—	1 (ref)	—
Substantially Increased	1.50 (1.47–1.52)	<0.001	1.89 (1.85–1.92)	<0.001	1.76 (1.70–1.80)	<0.001
*P* for trend		<0.001		<0.001		<0.001
ABSI and PhenoAge acceleration						
Q 1	1 (ref)	—	1 (ref)	—	1 (ref)	—
Q 2	1.22 (1.20–1.25)	<0.001	1.03 (1.01–1.05)	0.005	1.02 (1.00–1.05)	0.034
Q 3	1.57 (1.54–1.61)	<0.001	1.13 (1.10–1.15)	<0.001	1.11 (1.09–1.14)	<0.001
Q 4	2.08 (2.03–2.12)	<0.001	1.34 (1.31–1.37)	<0.001	1.30 (1.27–1.33)	<0.001
*P* for trend		<0.001		<0.001		<0.001
ABSI and KDMAge acceleration						
Q 1	1 (ref)	—	1 (ref)	—	1 (ref)	—
Q 2	1.09 (1.07–1.11)	<0.001	1.25 (1.22–1.30)	<0.001	1.22 (1.19–1.25)	<0.001
Q 3	1.03 (1.01–1.05)	<0.001	1.40 (1.37–1.43)	<0.001	1.34 (1.30–1.37)	<0.001
Q 4	1.15 (1.12–1.17)	<0.001	1.70 (1.65–1.74)	<0.001	1.58 (1.54–1.62)	<0.001
*P* for trend		<0.001		<0.001		<0.001
C-Index and PhenoAge acceleration						
Q 1	1 (ref)	—	1 (ref)	—	1 (ref)	—
Q 2	1.40 (1.37–1.43)	<0.001	1.22 (1.20–1.25)	<0.001	1.21 (1.19–1.24)	<0.001
Q 3	1.91 (1.87–1.95)	<0.001	1.50 (1.46–1.53)	<0.001	1.47 (1.44–1.51)	<0.001
Q 4	2.84 (2.78–2.90)	<0.001	2.08 (2.03–2.13)	<0.001	2.01 (1.96–2.06)	<0.001
*P* for trend		<0.001		<0.001		<0.001
C-Index and KDMAge acceleration						
Q 1	1 (ref)	—	1 (ref)	—	1 (ref)	—
Q 2	1.27 (1.24–1.29)	<0.001	1.50 (1.50–1.57)	<0.001	1.48 (1.45–1.52)	<0.001
Q 3	1.29 (1.26–1.32)	<0.001	1.91 (1.86–1.96)	<0.001	1.80 (1.75–1.85)	<0.001
Q 4	1.67 (1.63–1.70)	<0.001	2.76 (2.69–2.83)	<0.001	2.50 (2.44–2.57)	<0.001
*P* for trend		<0.001		<0.001		<0.001

Crude Model: Unadjusted.

Model 1: Adjusted for age, gender, race, Townsend deprivation index, education level, smoking status, alcohol drinker status.

Model 2: Base on Model2, further adjusted for history of hypertension/diabetes/hyperlipidemia/COPD, family history of lung cancer/diabetes/hypertension/hyperlipidemia and fasting time.

Abbreviations: WC (cm), waist circumference; WHR, waist–hip ratio; ABSI, a body shape index; C-Index, conicity index; KDMAge, Klemera-Doubal method biological age; PhenoAge, phenotypic age.

### 3.4 Association of accelerated biological aging with the risk of lung cancer

**FIGURE 2:**
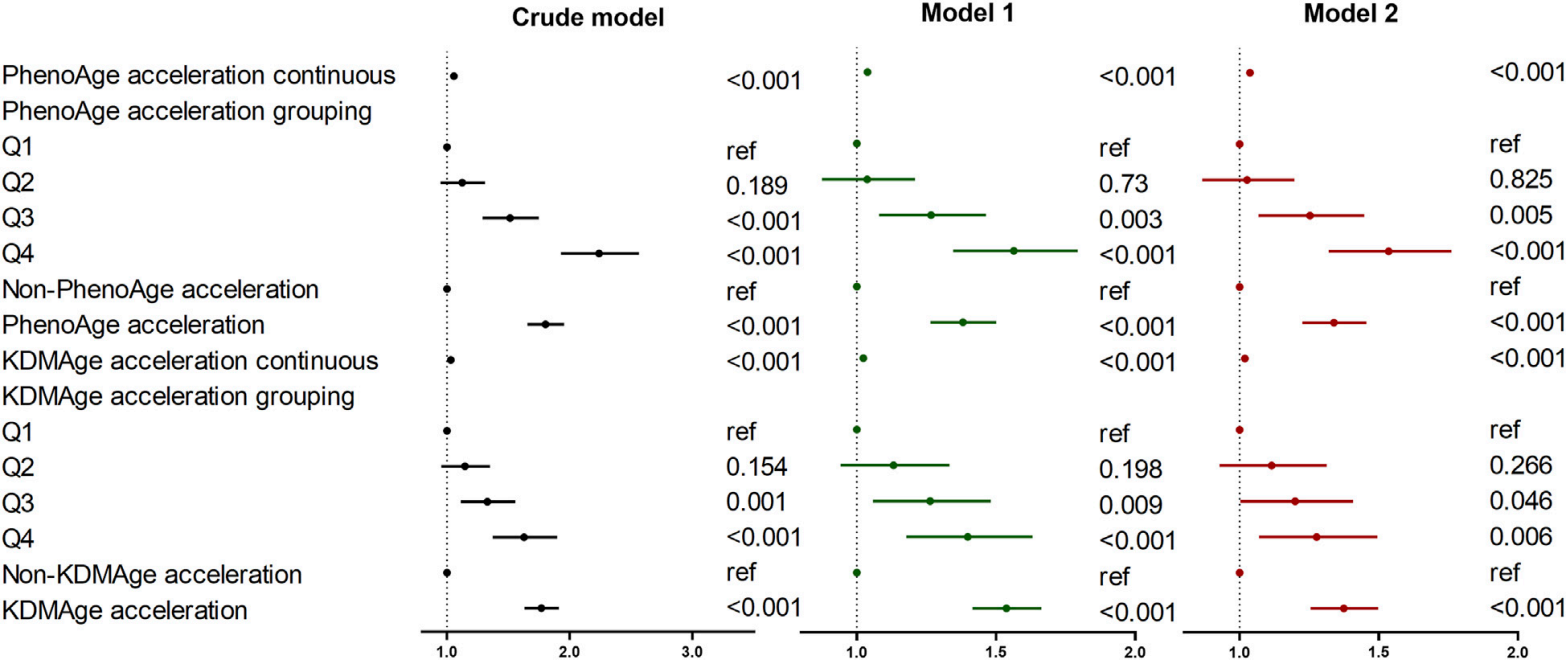
Associations of biological age measures with the risk of lung cancer. Crude model: Unadjusted. Model 1: Adjusted for age, gender, race, Townsend deprivation index, education level, smoking status, and alcohol drinker status. Model 2: Model 1 was further adjusted for history of hypertension/diabetes/hyperlipidemia/COPD, family history of lung cancer/diabetes/hypertension/hyperlipidemia and fasting time. Abbreviations: KDMAge, Klemera-Doubal method age, PhenoAge, phenotypic age.

The results of the multivariable regression analysis of the associations between accelerated biological aging and the risk of lung cancer are presented in Figure 2. The results indicate that in three Models, higher KDMAge acceleration and PhenoAge acceleration were all positively correlated with the risk of lung cancer. In the fully adjusted Model 2, participants in the highest quartile of KDMAge acceleration (HR = 1.27; 95% CI: 1.07–1.50) and PhenoAge acceleration (HR = 1.53; 95% CI:1.32–1.76) had higher lung cancer risk than those in the lowest quartiles. Additionally, a dose response relationship was still found when KDMAge acceleration and PhenoAge acceleration were converted from continuous variables to categorical variables (quartiles) for sensitivity analysis (Supplementary Figure S3).

### 3.5 Mediating role of accelerated biological aging in the association of central obesity with the risk of lung cancer

**FIGURE 3:**
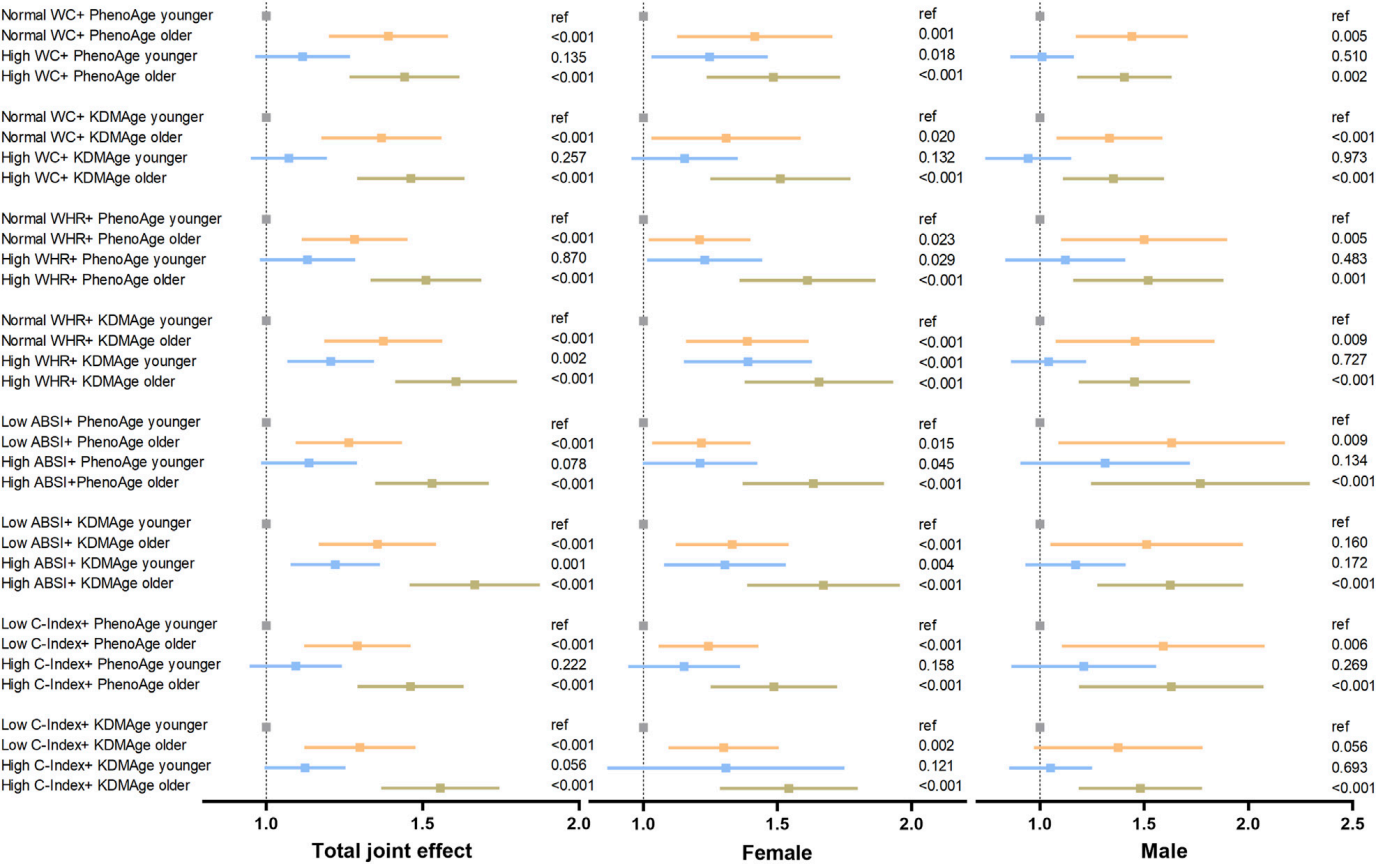
Joint effect of accelerated biological aging and central obesity-related indices on the risk of lung cancer. The models were adjusted for age, gender, race, education level, the Townsend deprivation index, smoking status, alcohol drinker status, history of hypertension, diabetes, hyperlipidaemia, COPD, family history of lung cancer/diabetes/hypertension/hyperlipidaemia and fasting time. The WC and WHR subgroups were classified by exceeding normal defined high levels, and the ABSI and C-Index subgroups were classified by median values. Abbreviations: WC (cm), waist circumference; WHR, waist–hip ratio; ABSI, body shape index; C-Index, conicity index; KDMAge, Klemera-Doubal method age; PhenoAge, phenotypic age.

**FIGURE 4:**
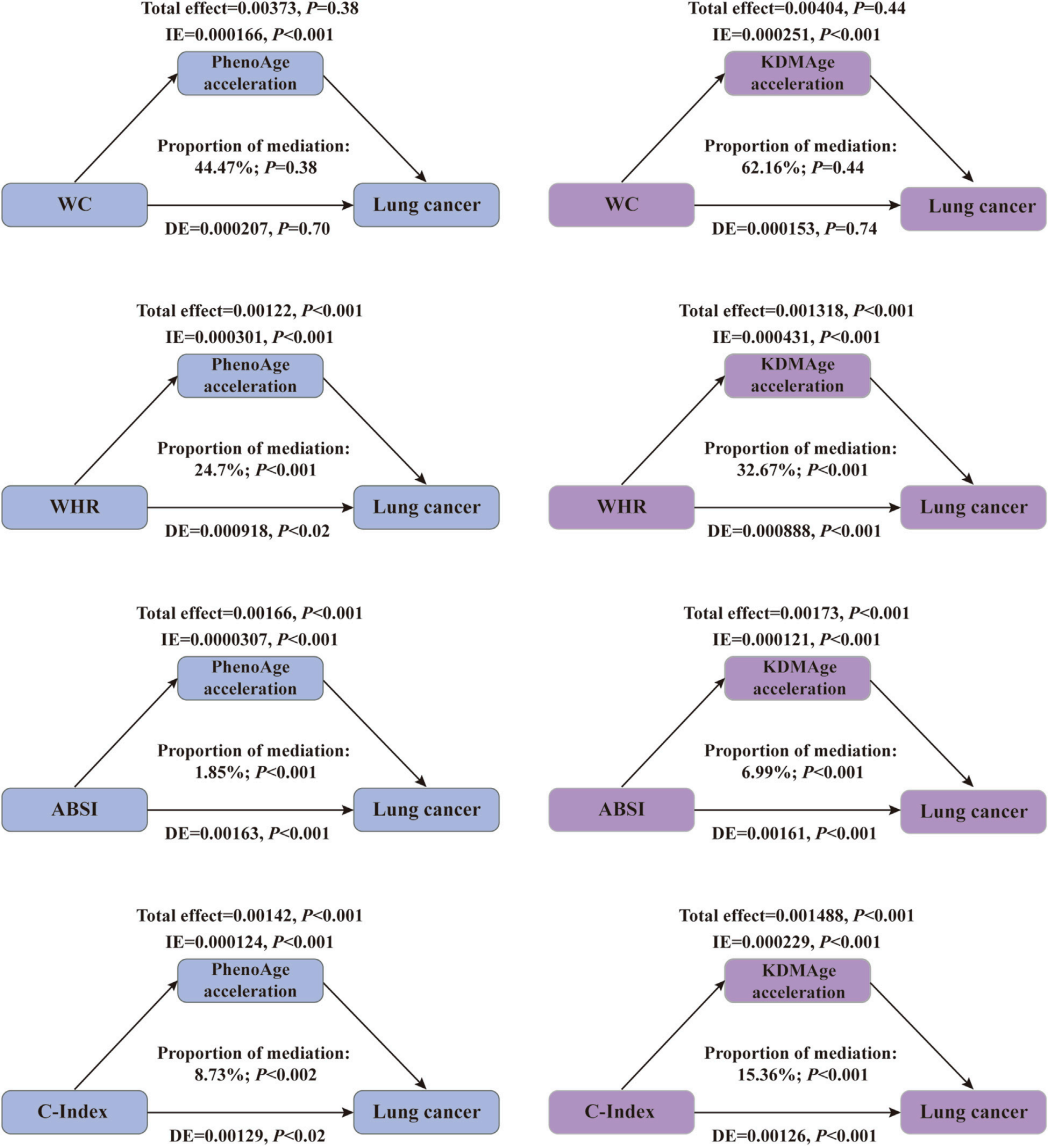
The mediating effect of accelerated biological aging on the association of central obesity with the risk of lung cancer. The models were adjusted for age, gender, race, education level, Townsend deprivation index, smoking status, alcohol drinker status, history of hypertension/diabetes/hyperlipidemia/COPD, family history of lung cancer/diabetes/hypertension/hyperlipidemia and fasting time. Abbreviations: KDMAge, Klemera–Doubal method age; WC (cm), waist circumference; WHR, waist‒hip ratio; ABSI, body shape index; C-Inedx, conicity index; PhenoAge, phenotypic age; IE, indirect effect; DE, direct effect.

Since central obesity and accelerated biological aging are potential lung cancer risk factors, we explored their joint and interactive effects. In most cases, a consistent upward trend in their combined impact on lung cancer risk was seen in Figure 3. For instance, compared to those with lower ABSI and younger KDMAge, individuals with high ABSI and older KDMAge had a 65.8% higher lung cancer risk. However, PhenoAge and KDMAge acceleration did not exhibit a significant positive additive interaction with central obesity in relation to the risk of lung cancer, as presented in Supplementary Table S1. Given that accelerated biological aging might serve as an intermediate link in the pathogenesis of central - obesity - induced lung cancer, we proceeded to conduct mediation analyses. Results indicated that both PhenoAge and KDMAge acceleration partially mediated the relationship between central obesity and incident lung cancer risk. Specifically, KDMAge or PhenoAge acceleration mediated the associations between WHR, ABSI, C - Index and the risk, with mediated proportions from 1.85% to 32.67% (all *P* < 0.05, Figure 4). Notably, no mediation effect of WC on lung cancer risk were observed (all *P* > 0.05).

### 3.6 Stratified and sensitivity analyses

Stratified analysis revealed that Gender significantly modified the associations between central obesity and the risk of incident lung cancer. Notably, these associations were far more conspicuous among females, as detailed in Supplementary Table S2. Moreover, smoking status significantly modulated the relationship between central obesity and the risk of incident lung cancer. Specifically, the association was particularly prominent among participants with a history of previous or current smoking, as presented in Supplementary Table S3. However, after a comprehensive examination, no significant modifying effects on the relationship between central obesity and the risk of incident lung cancer were identified. Consequently, the associations remained relatively consistent across various subgroups, as evidenced by Supplementary Tables S2, S3.

Sensitivity analyses demonstrated that the results were generally robust when excluding participants who had been diagnosed with lung cancer within the 2 years of enrollment were excluded to minimize the potential for reverse causality. To test model stability, pack years of smoking replaced traditional smoking status (Supplementary Tables S4, S5). The results were roughly similar to the above results.

## 4 Discussion

This study utilized the UK Biobank database to conduct a large-scale national prospective cohort study, aiming to systematically evaluate analyse the relationships among obesity, biological aging, and the risk of lung cancer in detail. From 2006 to 2010, the study ultimately included a total of 301,398 participants, among whom 159,405 were females, 141,993 were males, and 2,466 were diagnosed with lung cancer. Two BA measures, namely, KDMAge and PhenoAge, were employed in this research. Additionally, KDMAge acceleration and PhenoAge acceleration, both adjusted for chronological age, were analysed to comprehensively explore the associations among obesity, biological aging, and the risk of lung cancer. We found that, compared to people with obesity, people with central obesity presented significantly greater levels of KDMAge, PhenoAge, KDMAge acceleration, and PhenoAge acceleration, as well as a significantly increased risk of lung cancer. Mediation analysis indicated that the acceleration of KDMAge and PhenoAge accounted for 1.85%–32.67% of the total effect on the relationship between central obesity and lung cancer, suggesting a nonnegligible mediating role (*P* < 0.001).

The World Cancer Research Fund (WCRF), in collaboration with the International Agency for Research on Cancer (IARC), has estimated that approximately 20% of all cancer cases can be traced back to being overweight or obese (Tybjerg et al., 2022; Clinton et al., 2020). In the realm of clinical practice, BMI and WC have long been conventionally employed as key metrics for assessing obesity (Liang et al., 2021). However, BMI is not a reliable predictor of body fat distribution or metabolic status (Khan et al., 2023; Wee, 2022). This shortcoming has spurred further research into more effective assessment methods. Against this backdrop, Rodolfo Valdez and Krakauer independently proposed the C-Index and ABSI as novel tools for evaluating obesity (Krakauer and Krakauer, 2012; Valdez, 1991). These innovative indices stand in sharp contrast to traditional measures such as BMI and WC. The C-Index and ABSI have unique advantages: they are capable of comprehensively evaluating not only the overall fat mass inpeople with obesity but also the abdominal fat content in those with a leaner physique (Martins et al., 2023; Nkwana et al., 2021). This characteristic endows them with remarkable potential in unearthing latent central obesity cases. In light of the abovementioned advantages, in this particular research endeavor, we avoided the sole reliance on BMI. Instead, we opted to adopt a more comprehensive approach, integrating the WHR, C-Index, ABSI, and WC to assess central obesity precisely. The results clearly revealed a positive correlation between central obesity and the risk of lung cancer. This finding further validates the conclusions drawn from previous similar prospective cohort studies (Hidayat et al., 2016; Barbi et al., 2021). The possible mechanisms for this association include systemic chronic inflammation caused by central obesity. In people with obesity, the levels of inflammatory markers in the blood, such as CRP and white blood cell count, are elevated. On the other hand, pathophysiological studies have also demonstrated the potential role of chronic inflammatory factors in the development of lung cancer and in animal models induced by obesity (Vasiliki et al., 2024; Li et al., 2023). Moreover, oxidative stress triggered by smoking and environmental exposure, together with chronic inflammation resulting from central obesity, act synergistically to promote the growth and proliferation of lung cancer cells, further increasing the risk of developing lung cancer. However, although existing evidence indicates an association, the relationship between central obesity and the pathogenesis of lung cancer still requires in-depth research, and most of its underlying pathophysiological mechanisms remain unclear.

With increasing concern over the health impacts of obesity, an increasing number of studies have indicated that obesity could be a pivotal factor in accelerating biological aging (Cao et al., 2023; He et al., 2025). Moreover, weight loss interventions in people with obesity have been shown to lengthen telomere length, decrease DNA methylation, and improve functional age metrics (Cao et al., 2023; Mason et al., 2018; Petersen et al., 2021). Through multivariate regression analysis, this study revealed that in fully adjusted Model 2, the values of KDMAge, KDMAge acceleration, PhenoAge, and PhenoAge acceleration in the central obesity group were significantly greater than those in the noncentral obesity group (*P* < 0.001), and there was a dose‒response relationship (*p* for trend < 0.001). These results are consistent with previous research in U.S. adults that utilized telomere length, KDM-Age, and PhenoAge as markers of aging, lending strong support to the hypothesis that obesity promotes aging (He et al., 2025; Zhang et al., 2021; Santos and Sinha, 2021). In particular, central obesity not only promotes but also accelerates biological aging. Among the various factors underlying this connection, chronic low-grade inflammation is crucial. In people with obesity, especially those with central obesity, adipose tissue dysfunction, caused by factors such as increased immune cell infiltration, abnormal adipokine secretion, and heightened oxidative stress, triggers a state of chronic low-grade inflammation. During the process of organismal aging, cellular and molecular changes also lead to an increase in the production of proinflammatory cytokines, resulting in a similar chronic inflammatory state. This shared inflammatory state drives the development of pathophysiological processes such as insulin resistance, metabolic dysregulation, and immune dysfunction (Santos and Sinha, 2021; Trim et al., 2018), ultimately leading to age-related diseases, including type 2 diabetes, cardiovascular diseases, musculoskeletal disorders, and cancer (He et al., 2025; Barbe-Tuana et al., 2020; Wang et al., 2024; Zhao et al., 2024).

Aging is the most significant common risk factor for chronic diseases, including lung cancer. Most previous studies focused on the relationship between advanced chronological age and the incidence of lung cancer (Toumazis et al., 2020; Bates et al., 2022). In contrast, this study integrated multiple biomarkers as indicators of biological aging (reflecting the overall physiological status of an individual). The results revealed that greater biological age and biological aging acceleration were positively correlated with the risk of lung cancer (*P* < 0.001), and there was a dose–response relationship (*P* for trend < 0.001), indicating a significant association between the composite measures of biological aging and the risk of lung cancer. This finding is consistent with the literature, which has shown an association between increased epigenetic modifications and an increased risk of lung cancer (Dugue et al., 2021; Levine et al., 2015; Hillary et al., 2020). Taken together, these findings may indicate that multiple aging processes captured by biological aging measures (e.g., epigenetic alterations, inflammation, metabolic changes) could play a role in predicting cancer development. Although our biological aging algorithms included FEV1, a lung function biomarker strongly associated with lung cancer (Wasswa-Kintu et al., 2005), the original Levine PhenoAge algorithm (without FEV1) also showed a strong association with lung cancer (Levine et al., 2018), suggesting that the association between biological aging measures and lung cancer is not entirely due to decreased lung function. In addition, this association was significant only among ever-smokers, not among never-smokers, indicating that it may be confounded by smoking status, as smoking can lead to both advanced biological aging and an increased risk of lung cancer (Mamoshina et al., 2019). Therefore, the underlying mechanisms between biological aging and lung cancer still need further investigation.

Given that central obesity promotes biological aging and increases the risk of lung cancer and that biological aging also increases the risk of lung cancer, we further explored their joint or mediating effects on the incidence of lung cancer. Although there was no joint effect of KDMAge acceleration or PhenoAge acceleration with central obesity-related indices on the risk of lung cancer, generally, higher central obesity-related indices and biological age were associated with a greater risk of lung cancer. Chronic inflammation, particularly “inflammaging” (low-grade, persistent age-related inflammation), is a shared core mechanism for age-related diseases including lung cancer (Terron-Puig et al., 2022; Sanada et al., 2018). Central obesity, characterized by excessive visceral adipose tissue (VAT), amplifies inflammaging by exacerbating systemic inflammation and disrupting metabolic homeostasis, thereby creating a pro-tumorigenic microenvironment that favors lung cancer development (Ellulu et al., 2017). This link relies on the synergistic dysregulation of three aging-related pathways: mitochondrial dysfunction, senescence-associated secretory phenotype (SASP), and insulin/IGF-1 signaling (Terron-Puig et al., 2022; Sanada et al., 2018). VAT secretes pro-inflammatory adipokines (e.g., IL-6, TNF-α) that induce insulin resistance (a lung cancer risk factor (Rosario et al., 2022; Qi et al., 2023) and impair mitochondrial function. VAT-driven adipocyte expansion triggers mitochondrial stress and reactive oxygen species (ROS) overproduction: ROS damages lung epithelial cell DNA (promoting oncogenic mutations) and activates NF-κB, further amplifying inflammation and accelerating biological aging (Brunelli et al., 2021). VAT expansion also causes local hypoxia and adipocyte apoptosis, recruiting macrophages to form pro-inflammatory foci (Brunelli et al., 2021). This inflammation induces VAT cells and stromal cells to senesce, releasing SASP factors (e.g., IL-8, MMP-9). SASP sustains inflammaging and remodels the lung microenvironment (via angiogenesis and immune suppression), facilitating lung cancer progression (Yang et al., 2025). Additionally, obesity-induced insulin resistance disrupts insulin/IGF-1 signaling, thereby overactivating the PI3K/Akt/mTOR pathway. This pathway activation inhibits autophagy (which accelerates senescence) and enhances nutrient metabolism in lung cancer cells, ultimately promoting their proliferation and survival (Xu et al., 2025). Collectively, these pathways form a feedforward loop: central obesity drives the progression of inflammaging and biological aging, and these two processes in turn amplify lung cancer risk. This pattern aligns closely with the key finding of the present study, that biological aging acts as a mediator in the association between central obesity and lung cancer. Given this established mediating role, specific age-related processes underlying this pathway (e.g., metabolic dysregulation, as well as cellular functional decline such as impaired autophagy or mitochondrial dysfunction) merit further investigation. Notably, no mediating effect of biological aging acceleration or phenotypic age acceleration was detected on the association between WC and lung cancer risk. As a unidimensional indicator of trunk size, WC is unable to capture body fat distribution and body shape characteristics—unlike ratio-based anthropometric indices, including WHR, ABSI, and C-Index. These ratio-derived indices, by contrast, are more closely aligned with the mechanisms of metabolic dysfunction that underlie biological aging. Furthermore, WC fails to fully reflect metabolic abnormalities, which represent a core link connecting biological aging to lung cancer risk; this limitation may explain why WC did not exhibit a mediating effect in our analysis. Consistent with this reasoning, previous studies have also highlighted that WC cannot adequately characterize fat distribution or metabolic status (Lee et al., 2022; Wang et al., 2018), suggesting it may not be an optimal indicator for assessing central obesity in lung cancer research. In addition, KDMAge and PhenoAge differ in their ability to assess biological aging. KDMAge models biological age as the average biological state associated with a particular chronological age in a reference population, assuming a linear increase over time (Kwon and Belsky, 2021). PhenoAge models biological age as the average biological state associated with a specific level of mortality risk in a reference population, assuming an exponential increase over time. Genome-wide association studies have shown that genes associated with KDMAge are enriched in lipid-related pathways, whereas genes associated with PhenoAge are enriched in pathways related to the immune system, cell function, and carbohydrate homeostasis (Kuo et al., 2021). Moreover, the consistent results of the two aging scales in joint, interaction, and mediation analyses indicate that the accelerated biological aging caused by central obesity may jointly affect the development of lung cancer by interfering with lipid metabolism and the immune system. Intervening in central obesity might alleviate systemic inflammation, improve metabolic health, and slow biological aging, providing an effective approach to reduce the risk of lung cancer.

This study, as a large-scale national prospective cohort study, demonstrates notable strengths: it precisely adjusted for age as a core confounder when calculating biological age acceleration, verified the stability of core associations via rigorous subgroup, sensitivity, and stratified analyses, and explored the relationships among central obesity, biological age, and the risk of lung cancer and investigated the role of accelerated biological aging in this context, providing new clues for lung cancer prevention and treatment and filling research gaps. However, the study has prominent constraints on causal conclusions and generalizability: inherent observational data issues include ambiguous temporality (unclear measurement intervals between central obesity, biological age, and lung cancer may cause reverse causality, e.g., early lung cancer affecting biological age) and persistent residual confounding (unmeasured factors like metabolic/aging genetic susceptibility, smoking details, and chronic inflammation); the predominantly White British cohort severely restricts generalizability, as ethnic differences in central obesity (e.g., Asians’ lower BMI for visceral fat) and lung cancer pathogenesis (distinct risk exposures/subtypes) mean results cannot extend to other ethnic groups; additionally, there are methodological issues including information bias from questionnaires and self-reported variables, sample selection bias from analyzing only a cohort subset, inaccurate central obesity assessment due to lack of objective methods (e.g., CT/MRI), and inability to explore ethnic heterogeneity in subgroup analyses due to insufficient sample sizes. To address these, future studies should adopt repeated measurements to clarify temporality, incorporate more objective confounders and advanced analytical methods to reduce residual confounding, enhance ethnic diversity via multi-ethnic databases or cohorts, optimize variable measurement with objective tools, and avoid selection bias through clear subset criteria or full-cohort analysis.

## 5 Conclusion

In conclusion, this study demonstrated that central obesity can promote biological aging and increase the risk of lung cancer, and quantified the mediating effect of accelerated biological aging in this process.

## Data Availability

The original contributions presented in the study are included in the article/Supplementary Material, further inquiries can be directed to the corresponding author.
