# Supplementary material for: Accelerated biological aging as potential mediator in the relationship between central obesity and lung cancer risk
Source: Front Aging. 2025 Sep 19;6:1667490. doi: 10.3389/fragi.2025.1667490 (PMC12491821; doi:10.3389/fragi.2025.1667490)
Supplement: Supplementary file 1 [file Table1.docx]

# Supplementary Methods

# Biological age (BioAge)

$$BioAge=\frac{\sum_{i=1}^{n} \left( x_{i}-q_{i} \right)\frac{k_{i}}{s_{i}^{2}}+\frac{Chronological Age}{s_{BA}^{2}}}{\sum_{i=1}^{n} {(\frac{k_{i}}{s_{i}})}^{2}+\frac{1}{s_{BA}^{2}}}$$

where *x* is the value of biomarker *i* measured for an individual. The parameters *k*, *q*, and *s* denote intercept, slope, and root mean squared error, respectively, from a regression estimate of chronological age on the biomarker in the reference sample. *s_BA_* denotes a scaling factor equal to the square root of the variance in chronological age explained by the biomarker set in the reference sample.

# Phenotypic age (PhenoAge)

$$PhenoAge=141.50+\frac{\ln\left[ -0.00553\times ln \left( 1-M \right) \right]}{0.09165}$$

$$M=1-exp(\frac{-1.51714\times exp(xb)}{0.007250078})$$

$$xb = -19.907 - 0.0336 \times Albumin + 0.0095 \times Creatinine + 0.1953 \times Glucose$$

$$+ 0.0954 \times ln(CRP) - 0.0120 \times Lymphocyte Percent$$

$$+ 0.0268 \times Mean Cell Volume + 0.3306 \times Red Cell Distribution Width$$

$$+ 0.00188 \times Alkaline Phosphatase + 0.0554 \times White Blood Cell Count$$

$$+ 0.0804 \times Chronological Age$$
