# Supplementary figures and images for: Accelerated biological aging as potential mediator in the relationship between central obesity and lung cancer risk
Source: Front Aging. 2025 Sep 19;6:1667490. doi: 10.3389/fragi.2025.1667490 (PMC12491821; doi:10.3389/fragi.2025.1667490)

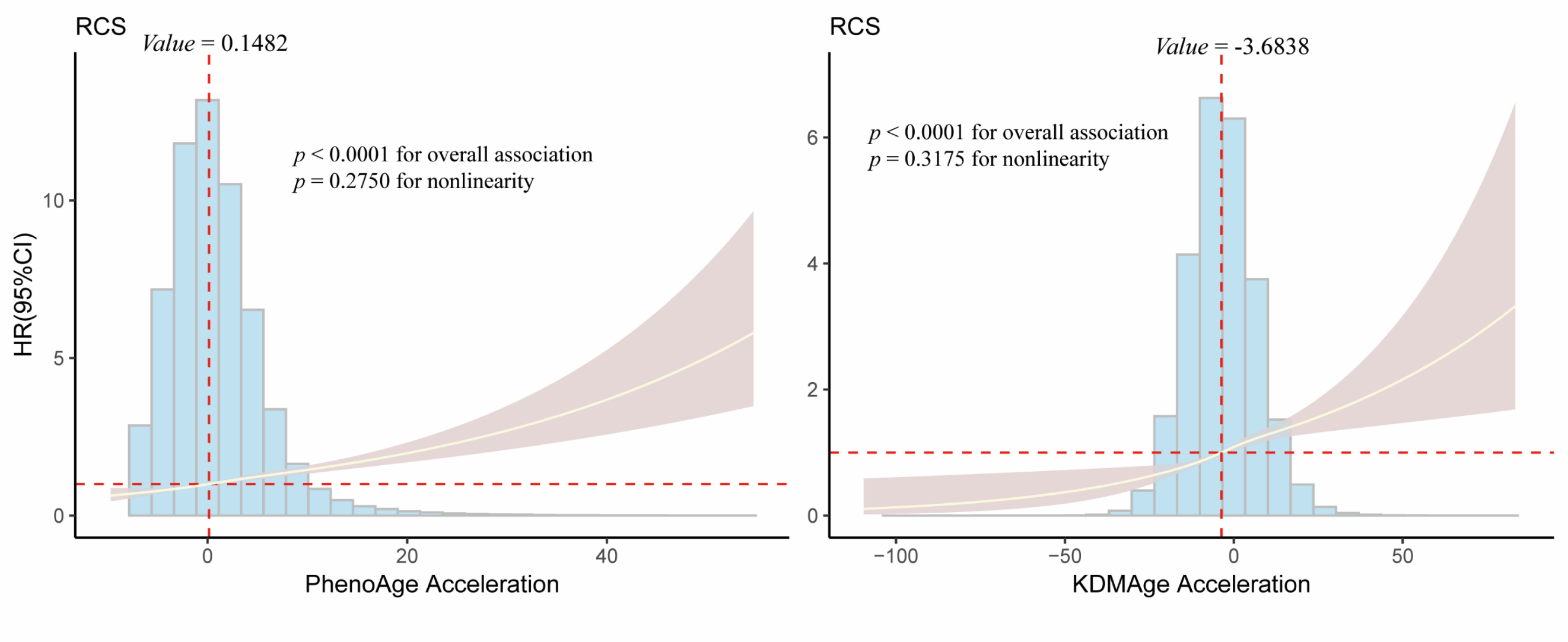

Supplement: Supplementary file 2 [file Image3.tif]

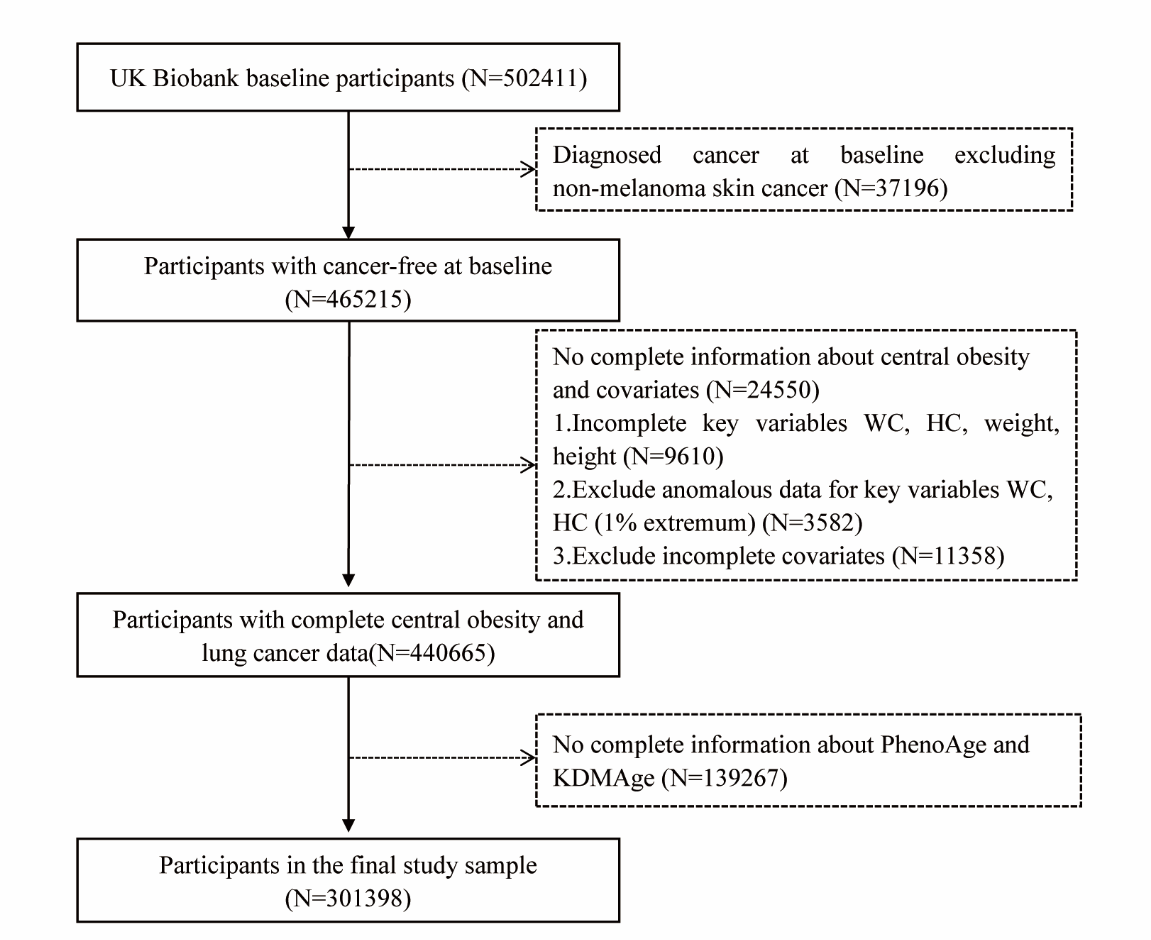

Supplement: Supplementary file 4 [file Image1.tif]
