# Supplementary material for: Accelerated biological aging as potential mediator in the relationship between central obesity and lung cancer risk
Source: Front Aging. 2025 Sep 19;6:1667490. doi: 10.3389/fragi.2025.1667490 (PMC12491821; doi:10.3389/fragi.2025.1667490)
Supplement: Supplementary file 5 [file Table2.docx]

**Table S1 Interaction between central obesity and accelerated biological aging on lung cancer risk**

|  | **Multiplicative interaction** | | **Additive interaction** | |
| --- | --- | --- | --- | --- |
|  | **HR (95% CI)** | ***P* value** | **RERI (95% CI)** | ***P* value** |
| **PhenoAge acceleration** | | | | |
| WC | 0.94 (0.79, 1.11) | 0.459 | -0.05 (-0.26, 0.15) | 0.698 |
| WHR | 1.05 (0.88, 1.24) | 0.588 | 0.11 (-0.09, 0.31) | 0.137 |
| ABSI | 1.08 (0.91, 1.28) | 0.406 | 0.16 (-0.04, 0.36) | 0.063 |
| C-Index | 1.04 (0.88, 1.24) | 0.640 | 0.09 (-0.11, 0.29) | 0.184 |
| **KDMAge acceleration** | | | | |
| WC | 1.00 (0.85, 1.19) | 0.968 | 0.03 (-0.18, 0.23) | 0.390 |
| WHR | 0.97 (0.83, 1.15) | 0.763 | 0.03 (-0.18, 0.24) | 0.399 |
| ABSI | 1.01 (0.86, 1.20) | 0.874 | 0.10 (-0.12, 0.31) | 0.190 |
| C-Index | 1.07 (0.91, 1.27) | 0.400 | 0.13 (-0.07, 0.33) | 0.094 |

The models were adjusted for age, sex, race, education levels, Townsend deprivation index, smoking status, alcohol drinker status, history of hypertension, diabetes, hyperlipidemia, COPD, family history of lung cancer/diabetes/ hypertension/hyperlipidemia and fasting time. WC and WHR subgroups were classified by exceed normal defined high levels, ABSI and C-Index subgroups were classified by median values. Abbreviations: WC, waist circumference; WHR, waist–hip ratio; ABSI, a body shape index; C-Index, conicity index; KDMAge, Klemera-Doubal method biological age; PhenoAge, phenotypic age.

**Table S2 Risk analysis of the association between central obesity - related indices and lung cancer under gender stratification**

|  | Female | | Male | |
| --- | --- | --- | --- | --- |
|  | HR (95%CI) | P value | HR (95%CI) | P value |
| **WC and Lung cancer** | | | | |
| Normal | 1 (ref) | .. | 1 (ref) | .. |
| Increased | 1.06 (0.94-1.20) | 0.356 | 0.99(0.89−1.10) | 0.842 |
| Substantially Increased | 1.17 (1.05-1.31) | 0.005 | 1.02(0.92−1.13) | 0.766 |
| **WHR and Lung cancer** | | | | |
| Normal | 1 (ref) | .. | 1 (ref) | .. |
| Substantially Increased | 1.26 (1.15-1.39) | <0.001 | 1.00(0.91−1.09) | 0.969 |
| **ABSI and lung cancer** | | | | |
| Q 1 | 1 (ref) | .. | 1 (ref) | .. |
| Q 2 | 1.30 (1.15-1.47) | <0.001 | 1.30(0.91−1.86) | 0.149 |
| Q 3 | 1.45 (1.27-1.66) | <0.001 | 1.32(0.94−1.87) | 0.109 |
| Q 4 | 1.71 (1.48-1.98) | <0.001 | 1.70(1.21−2.39) | 0.002 |
| **C-Index and lung cancer** | | | | |
| Q 1 | 1 (ref) | .. | 1 (ref) | .. |
| Q 2 | 1.24 (1.10-1.41) | <0.001 | 1.05(0.77−1.43) | 0.773 |
| Q 3 | 1.28 (1.12-1.46) | <0.001 | 1.06(0.79−1.43) | 0.699 |
| Q 4 | 1.49 (1.29-1.71) | <0.001 | 1.34(1.00−1.79) | 0.051 |

Crude model: Unadjusted.

Model 1: Adjusted for age, gender, race, Townsend deprivation index, education level, pack years of smoking, alcohol drinker status.

Model 2: Base on Model1, further adjusted for history of hypertension/diabetes/ hyperlipidemia/COPD, family history of lung cancer/diabetes/hypertension/hyperlipidemia.

**Table S3 Risk analysis of the association between central obesity - related indices and lung cancer under smoking status stratification**

|  | **Smoking status（Never）** | | **Smoking status（Previous）** | | **Smoking status（Current）** | |
| --- | --- | --- | --- | --- | --- | --- |
|  | **HR (95%CI)** | ***P* value** | **HR (95%CI)** | ***P* value** | **HR (95%CI)** | ***P* value** |
| **WC and Lung cancer** | | | | | | |
| Normal | 1 (ref) | .. | 1 (ref) | .. | 1 (ref) | .. |
| Increased | 0.93(0.75-1.15) | 0.48 | 1.14(1.00-1.29) | 0.044 | 0.96(0.85-1.09) | 0.522 |
| Substantially Increased | 1.28(1.05-1.55) | 0.01 | 1.26(1.12-1.41) | <0.001 | 0.94(0.83-1.06) | 0.294 |
| **WHR and Lung cancer** | | | | | | |
| Normal | 1 (ref) | .. | 1 (ref) | .. | 1 (ref) | .. |
| Substantially Increased | 1.08(0.90-1.28) | 0.42 | 1.165(1.06-1.28) | 0.002 | 1.05(0.95-1.16) | 0.323 |
| **ABSI and lung cancer** | | | | | | |
| Q 1 | 1 (ref) | .. | 1 (ref) | .. | 1 (ref) | .. |
| Q 2 | 1.09(0.86-1.38) | 0.46 | 1.38(1.17-1.64) | <0.001 | 1.33(1.09-1.63) | <0.001 |
| Q 3 | 1.11(0.86-1.43) | 0.43 | 1.36(1.14-1.62) | <0.001 | 1.61(1.32-1.97) | <0.001 |
| Q 4 | 1.14(0.86-1.50) | 0.36 | 1.75(1.47-2.09) | <0.001 | 2.07(1.70-2.52) | <0.001 |
| **C-Index and lung cancer** | | | | | | |
| Q 1 | 1 (ref) | .. | 1 (ref) | .. | 1 (ref) | .. |
| Q 2 | 1.14(0.91-1.44) | 0.26 | 1.24(1.05-1.48) | 0.013 | 1.20(1.00-1.44) | 0.050 |
| Q 3 | 0.88(0.68-1.151) | 0.36 | 1.33(1.12-1.58) | 0.001 | 1.28(1.06-1.54) | 0.009 |
| Q 4 | 1.16(0.89-1.52) | 0.28 | 1.66(1.40-1.97) | <0.001 | 1.53(1.28-1.85) | <0.001 |

Crude model: Unadjusted.

Model 1: Adjusted for age, gender, race, Townsend deprivation index, education level, pack years of smoking, alcohol drinker status.

Model 2: Base on Model1, further adjusted for history of hypertension/diabetes/ hyperlipidemia/COPD, family history of lung cancer/diabetes/hypertension/hyperlipidemia.

**Table S4. Association between central obesity and lung cancer after excluding participants with less than 2 years of follow-up**

|  | Crude model | | Model 1 | | Model 2 | |
| --- | --- | --- | --- | --- | --- | --- |
|  | **HR (95%CI)** | ***P* value** | **HR (95%CI)** | ***P* value** | **HR (95%CI)** | ***P* value** |
| WC and Lung cancer | | | | | | |
| Normal | 1 (ref) | .. | 1 (ref) | .. | 1 (ref) | .. |
| Increased | 1.23(1.13-1.34) | <0.001 | 0.98(0.90-1.07) | 0.691 | 1.03(0.95-1.12) | 0.449 |
| Substantially Increased | 1.50(1.38-1.62) | <0.001 | 1.11(1.02-1.20) | 0.013 | 1.17(1.08-1.27) | 0.001 |
| WHR and Lung cancer | | | | | | |
| Normal | 1 (ref) | .. | 1 (ref) | .. | 1 (ref) | .. |
| Substantially Increased | 1.44(1.34-1.54) | <0.001 | 1.11(1.04-1.19) | 0.002 | 1.13(1.05-1.21) | <0.001 |
| ABSI and lung cancer | | | | | | |
| Q 1 | 1 (ref) | .. | 1 (ref) | .. | 1 (ref) | .. |
| Q 2 | 1.59(1.41-1.79) | <0.001 | 1.30(1.15-1.47) | <0.001 | 1.31(1.16-1.48) | <0.001 |
| Q 3 | 1.93(1.72-2.17) | <0.001 | 1.39(1.20-1.57) | <0.001 | 1.40(1.24-1.58) | <0.001 |
| Q 4 | 3.38(3.03-3.76) | <0.001 | 1.86(1.65-2.10) | <0.001 | 1.77(1.57-2.00) | <0.001 |
| C-Index and Lung cancer | | | | | | |
| Q 1 | 1 (ref) | .. | 1 (ref) | .. | 1 (ref) | .. |
| Q 2 | 1.53(1.36-1.71) | <0.001 | 1.21(1.08-1.36) | 0.001 | 1.22(1.08-1.37) | 0.001 |
| Q 3 | 1.72(1.54-1.93) | <0.001 | 1.19(1.06-1.34) | 0.004 | 1.20(1.07-1.36) | 0.003 |
| Q 4 | 3.00(2.71-3.33) | <0.001 | 1.56(1.39-1.76) | <0.001 | 1.56(1.39-1.76) | <0.001 |

Crude model: Unadjusted.

Model 1: Adjusted for age, gender, race, Townsend deprivation index, education level, pack years of smoking, alcohol drinker status.

Model 2: Base on Model1, further adjusted for history of hypertension/diabetes/ hyperlipidemia/COPD, family history of lung cancer/diabetes/hypertension/hyperlipidemia.

**Table S5. Association between central obesity and lung cancer adjusting for pack years of smoking instead of smoking status**

|  | Crude model | | Model 1 | | Model 2 | |
| --- | --- | --- | --- | --- | --- | --- |
|  | **HR (95%CI)** | ***P* value** | **HR (95%CI)** | ***P* value** | **HR (95%CI)** | ***P* value** |
| WC and Lung cancer | | | | | | |
| Normal | 1 (ref) | .. | 1 (ref) | .. | 1 (ref) | .. |
| Increased | 1.24(1.15-1.34) | <0.001 | 0.95(0.87-1.03) | 0.173 | 0.98(0.91-1.06) | 0.655 |
| Substantially Increased | 1.49(1.39-1.61) | <0.001 | 0.90(0.83-0.96) | 0.004 | 0.94(0.87-1.01) | 0.091 |
| WHR and Lung cancer | | | | | | |
| Normal | 1 (ref) | .. | 1 (ref) | .. | 1 (ref) | .. |
| Substantially Increased | 1.91(1.79-2.04) | <0.001 | 1.15(1.07-1.24) | <0.001 | 1.17(1.09-1.26) | <0.001 |
| ABSI and lung cancer | | | | | | |
| Q 1 | 1 (ref) | .. | 1 (ref) | .. | 1 (ref) | .. |
| Q 2 | 1.59(1.43-1.78) | <0.001 | 1.36(1.21-1.52) | <0.001 | 1.37(1.22-1.53) | <0.001 |
| Q 3 | 1.97(1.77-2.19) | <0.001 | 1.47(1.31-1.65) | <0.001 | 1.48(1.32-1.66) | <0.001 |
| Q 4 | 3.39(3.07-3.74) | <0.001 | 1.84(1.64-2.07) | <0.001 | 1.77(1.57-1.98) | <0.001 |
| C-Index and Lung cancer | | | | | | |
| Q 1 | 1 (ref) | .. | 1 (ref) | .. | 1 (ref) | .. |
| Q 2 | 1.54(1.38-1.71) | <0.001 | 1.25(1.12-1.40) | 0.001 | 1.26(1.13-1.41) | <0.001 |
| Q 3 | 1.78(1.60-1.98) | <0.001 | 1.23(1.10-1.38) | 0.004 | 1.26(1.13-1.41) | <0.001 |
| Q 4 | 3.04(2.76-3.35) | <0.001 | 1.42(1.27-1.59) | <0.001 | 1.42(1.27-1.60) | <0.001 |

Crude model: Unadjusted.

Model 1: Adjusted for age, gender, race, Townsend deprivation index, education level, pack years of smoking, alcohol drinker status.

Model 2: Base on Model1, further adjusted for history of hypertension/diabetes/ hyperlipidemia/COPD, family history of lung cancer/diabetes/hypertension/hyperlipidemia.

Abbreviations: WC, waist circumference; WHR, waist–hip ratio; ABSI, a body shape index; C-Index, conicity index.
